# Environmental Shaping of Sponge Associated Archaeal Communities

**DOI:** 10.1371/journal.pone.0015774

**Published:** 2010-12-30

**Authors:** Aline S. Turque, Daniela Batista, Cynthia B. Silveira, Alexander M. Cardoso, Ricardo P. Vieira, Fernando C. Moraes, Maysa M. Clementino, Rodolpho M. Albano, Rodolfo Paranhos, Orlando B. Martins, Guilherme Muricy

**Affiliations:** 1 Instituto de Bioquímica Médica, Universidade Federal do Rio de Janeiro, Rio de Janeiro, Brazil; 2 Laboratório de Estudos Marinhos e Ambientais, Departamento de Química, Pontifícia Universidade Católica do Rio de Janeiro, Rio de Janeiro, Brazil; 3 Inmetro. Diretoria de Programa, Instituto Nacional de Metrologia Normalização e Qualidade Industrial, Rio de Janeiro, Brazil; 4 Departamento de Invertebrados, Museu Nacional, Universidade Federal do Rio de Janeiro, Rio de Janeiro, Brazil; 5 Laboratório de Microrganismos de Referência, INCQS, Fundação Oswaldo Cruz, Rio de Janeiro, Brazil; 6 Departamento de Bioquímica, Universidade do Estado do Rio de Janeiro, Rio de Janeiro, Brazil; 7 Departamento de Biologia Marinha, Instituto de Biologia, Universidade Federal do Rio de Janeiro, Rio de Janeiro, Brazil; Biodiversity Insitute of Ontario - University of Guelph, Canada

## Abstract

**Background:**

Archaea are ubiquitous symbionts of marine sponges but their ecological roles and the influence of environmental factors on these associations are still poorly understood.

**Methodology/Principal Findings:**

We compared the diversity and composition of archaea associated with seawater and with the sponges *Hymeniacidon heliophila, Paraleucilla magna* and *Petromica citrina* in two distinct environments: Guanabara Bay, a highly impacted estuary in Rio de Janeiro, Brazil, and the nearby Cagarras Archipelago. For this we used metagenomic analyses of 16S rRNA and ammonia monooxygenase (*amoA*) gene libraries. *Hymeniacidon heliophila* was more abundant inside the bay, while *P. magna* was more abundant outside and *P. citrina* was only recorded at the Cagarras Archipelago. Principal Component Analysis plots (PCA) generated using pairwise unweighted UniFrac distances showed that the archaeal community structure of inner bay seawater and sponges was different from that of coastal Cagarras Archipelago. Rarefaction analyses showed that inner bay archaeaoplankton were more diverse than those from the Cagarras Archipelago. Only members of *Crenarchaeota* were found in sponge libraries, while in seawater both *Crenarchaeota* and *Euryarchaeota* were observed. Although most *amoA* archaeal genes detected in this study seem to be novel, some clones were affiliated to known ammonia oxidizers such as *Nitrosopumilus maritimus* and *Cenarchaeum symbiosum*.

**Conclusion/Significance:**

The composition and diversity of archaeal communities associated with pollution-tolerant sponge species can change in a range of few kilometers, probably influenced by eutrophication. The presence of archaeal *amoA* genes in Porifera suggests that *Archaea* are involved in the nitrogen cycle within the sponge holobiont, possibly increasing its resistance to anthropogenic impacts. The higher diversity of *Crenarchaeota* in the polluted area suggests that some marine sponges are able to change the composition of their associated archaeal communities, thereby improving their fitness in impacted environments.

## Introduction

Sponges are ancient, sessile, highly efficient filter-feeding animals, with fossils dating back to the Late Precambrian [1]. Symbiont microbial communities are likely to have appeared in the same period, thus sharing a long association history with their sponge hosts [2]. Microbial associations are widespread in marine benthic invertebrates, but little is known about their physiological and ecological importance for the hosts. Sponges are among the invertebrate phyla that most commonly harbors associated microbial communities, and some species have even been called “bacteriosponges” due to the high content of bacterial cells in their tissues [3]. These symbiotic relationships occur with a variety of heterotrophic and autotrophic bacteria, archaea, protists and microalgae [2]. The evolutionary and ecological success obtained by Porifera may be in part related to this intimate association with microbial symbionts, in accordance to the hologenome theory that considers the host and its microbiota as a single evolutionary unit [4]. In fact, many symbiotic archaea found in sponges appear to be distinct from those present in seawater, marine sediment and plankton. Furthermore, the analysis of marine sponge microbial consortia has shown that sponges from different oceans contain specific microbial signatures [5]–[7].

*Archaea* are generally divided into two main phylogenetic lineages: *Crenarchaeota* and *Euryarchaeota*. Marine sponge associated archaea belong mainly to the *Crenarchaeota* phylum [8], but *Euryarchaeota* have also been documented in a few species [9], [10]. As most microbes associated with marine sponges are not amenable to cultivation techniques, their identity has been retrieved mainly by molecular techniques such as 16S rRNA gene libraries and metagenomics [5], [7], [9], [11].

Studies with the first cultivated non-thermophilic *Crenarchaeota*, *Nitrosopumilus maritimus,* isolated from marine aquarium sediment, demonstrate that bicarbonate and ammonia can serve as carbon and energy sources for some members of this autotrophic lineage [12]. Interestingly, this *Archaea* species is also associated with marine sponges. *Cenarchaeum symbiosum,* another species of the ubiquitous and abundant group of marine *Crenarchaeota*, is the sole archaeal symbiont of the marine sponge *Axinella mexicana* [13]. Although uncultivated, *C*. *symbiosum* can be harvested in significant quantities from sponge tissues for genomic studies [14]. Fosmid libraries have been constructed and the complete genome was assembled from enriched preparations of *C. symbiosum* DNA. The full genome sequences from these two mesophilic *Crenarchaeota* provide a new perspective in the study of sponge symbionts, their predicted metabolic pathways, population biology and gene representation in environmental *Archaea* surveys.

It has been suggested that sponge associated archaea may be involved in ammonia oxidation [15] performed by the enzyme ammonia monooxygenase, which catalyses ammonia oxidation to hydroxylamine. The occurrence of ammonia monooxygenase in environmental samples can be estimated by amplification of the *amoA* gene, which encodes the enzyme's catalytic subunit [16]. Ammonia oxidizing microbes play an important role in the global nitrogen cycle and also in marine invertebrate holobiont systems [4], [7], [15], [16], [17]. Sponges, for example, ingest organic bound nitrogen with their food and excrete ammonia as a metabolic end product [18]. In this regard, ammonia oxidizing microorganisms may be important in detoxifying sponge tissues. Symbiosis with ammonia oxidizing microorganisms may increase the fitness of their invertebrate host in polluted areas around large cities, which often contain high concentrations of ammonia. Although marine sponges mainly inhabit regions of oligotrophic seawater, they are also found in some polluted environments. Guanabara Bay is a highly eutrophic estuary in Rio de Janeiro, Brazil. Alterations in the drainage basin, petroleum and sewage pollution, and increased industrial output have led to severe environmental degradation with a marked decrease in water quality [19]. This resulted in increased eutrophic conditions, high sedimentation rates [20], elevated concentrations of toxic metals and hydrocarbons [21] and, consequently, many alterations in pelagic and benthic communities. Low salinity and high ammonia and phosphate concentrations are typically found inside the bay [22].

On the other hand, the Cagarras Archipelago, situated approximately 8 km Southwest from Guanabara Bay entrance, is a less impacted area. Composed of three islands (Cagarra, Palmas and Comprida) and four islets, the archipelago has recently been raised to a more restrictive category of conservation unit. Thus, ecological studies are needed to evaluate the response of the biota to future environmental management. These islands are impacted both by the Guanabara Bay waters and by discharges from a submarine outfall dumping untreated domestic sewage, which is balanced by pristine offshore water masses [23].

To date, metagenomic surveys of microorganisms associated with Southwestern Atlantic sponges have been restricted to bacteria and fungiae with archaea being totally neglected [24]–[27]. To better understand the influence of environmental factors on sponge associated microbial communities, we performed a survey of archaeal communities of the sponge species *Hymeniacidon heliophila* and *Paraleucilla magna.* We compared specimens occurring within Guanabara Bay (site P92) with those of the Cagarras Archipelago (CA). We also analyzed archaeal communities of *Petromica citrina* collected in the Cagarras Archipelago, as an example of a sponge species that is absent from Guanabara Bay. In addition, we analyzed the archaeal *amoA* gene distribution and phylogeny in these sponges to investigate their possible role in helping the host thrive in eutrophic areas. In this study, sponge associated archaea were analyzed for the first time in the Southwestern Atlantic Ocean and in a calcareous sponge through metagenomics. We demonstrate that each species has a distinct archaeal community and that species displaying diverse archaeal communities survive in the eutrophic environment

## Results

### Seawater chemistry and microbiology

**Figure 1:**
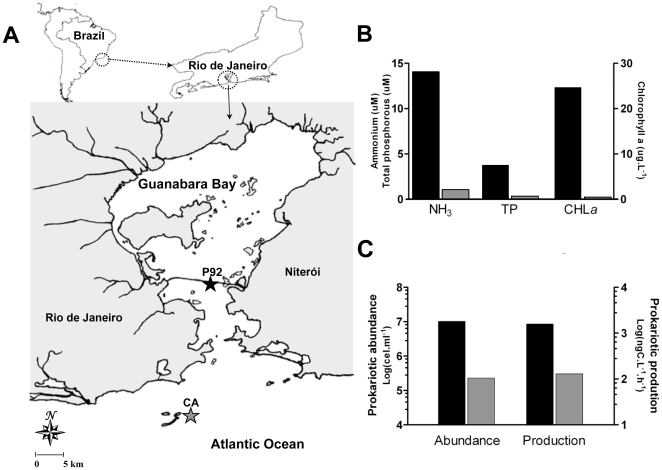
Location of sampling sites, seawater trophic status and planktonic microbiological parameters in Rio de Janeiro. (A) The location of Guanabara Bay in South America is indicated on the map (upper left corner). The map on the upper right corner shows the location of Guanabara Bay in reference to Rio de Janeiro state. The lower panel shows a detailed map of Guanabara Bay and the location of the two sampling sites: the pillar 92 of the Rio-Niterói Bridge, the inner bay site (P92) and the Cagarras Archipelago (CA), the outer bay site. (B) Ammonium, phosphate and chlorophyll *a* concentrations in seawater inside (black bars) and outside Guanabara Bay (gray bars). (C) Planktonic prokaryotic abundance and production inside (black bars) and outside the bay (gray bars).

To determine how archaeoplankton and sponge archaeal communities are linked to environmental conditions, water samples were collected at the two distinct sites (Fig. 1A). Abiotic and microbiological parameters at each site characterize two distinct water quality conditions. Phosphate and ammonium values were an order of magnitude higher within the bay than in the Cagarras Archipelago (Fig. 1B). The high levels of chlorophyll *a* underscore the eutrophic condition in bay waters. Bacterioplankton abundance was two orders of magnitude higher in bay waters (10^7^ cells.mL^−1^) compared to insular water (10^5^ cells.mL^−1^). Bacterial production was ten times higher at the inner bay site than in Cagarras Archipelago (Fig. 1C).

### Sponge morphology and ecology

**Figure 2:**
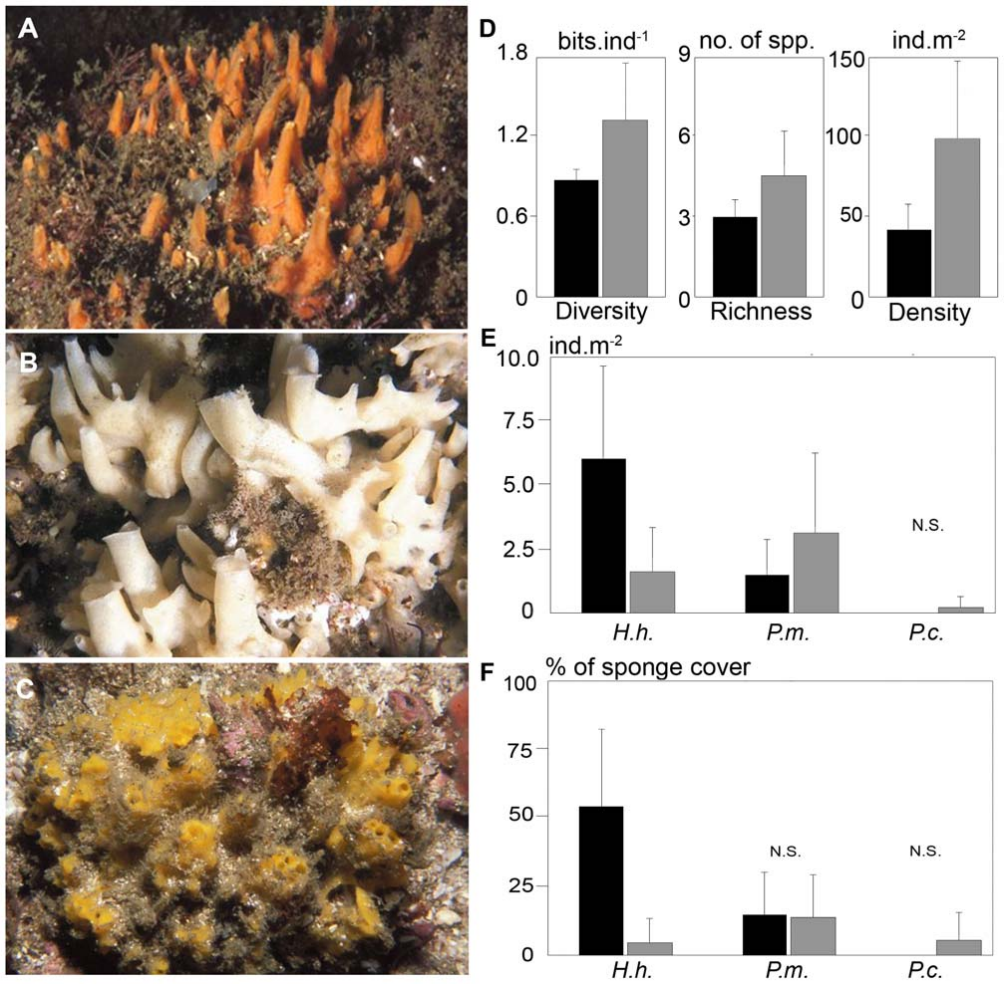
Sponge species, population and community structure. (A) *Hymeniacidon heliophila* (B) *Paraleucilla magna* (C) *Petromica citrina* (D) Indexes of whole sponge community structure: Shannon's diversity H' (bits per individual), species richness (number of species) and total sponge density (number of individuals per square meter) (E) Abundance of *H. heliophila* (*H.h*.), *P. magna* (*P.m.*) and *P. citrina* (*P.c*.) (number of individuals per square meter) (F) Dominance (% of total sponge cover) of *H. heliophila, P. magna* and *P. citrina* inside (black columns) and outside Guanabara Bay (gray columns). N.S., not significant. Error bars  =  standard deviation.

The three sponge species studied have similar sizes (approximately 10–20 cm long by 3–6 cm high) and all are thick encrusting to massive irregular, often forming upright projections topped by oscules (Fig. 2A–C; for detailed descriptions see [28]). All three species support relatively high sediment loads.

The sponge community within Guanabara Bay showed significantly lower species richness, diversity and density than at the Cagarras Archipelago (p<0.0002; Fig. 2D). *Paraleucilla magna* was more abundant outside the bay (p<0.0165) and *P. citrina* was completely absent in the inner bay site (Fig. 2E–F). In contrast, *H. heliophila* abundance more than doubled in the inner bay site as compared to the coastal site (p = 0.0001; Fig. 2E). As a result, there was a dominance of *P. magna* and *H. heliophila* inside the bay, reflecting their greater resistance to eutrophication compared to the other sponge species (Fig. 2F).

### Archaeoplankton biodiversity analyses

**Figure 3:**
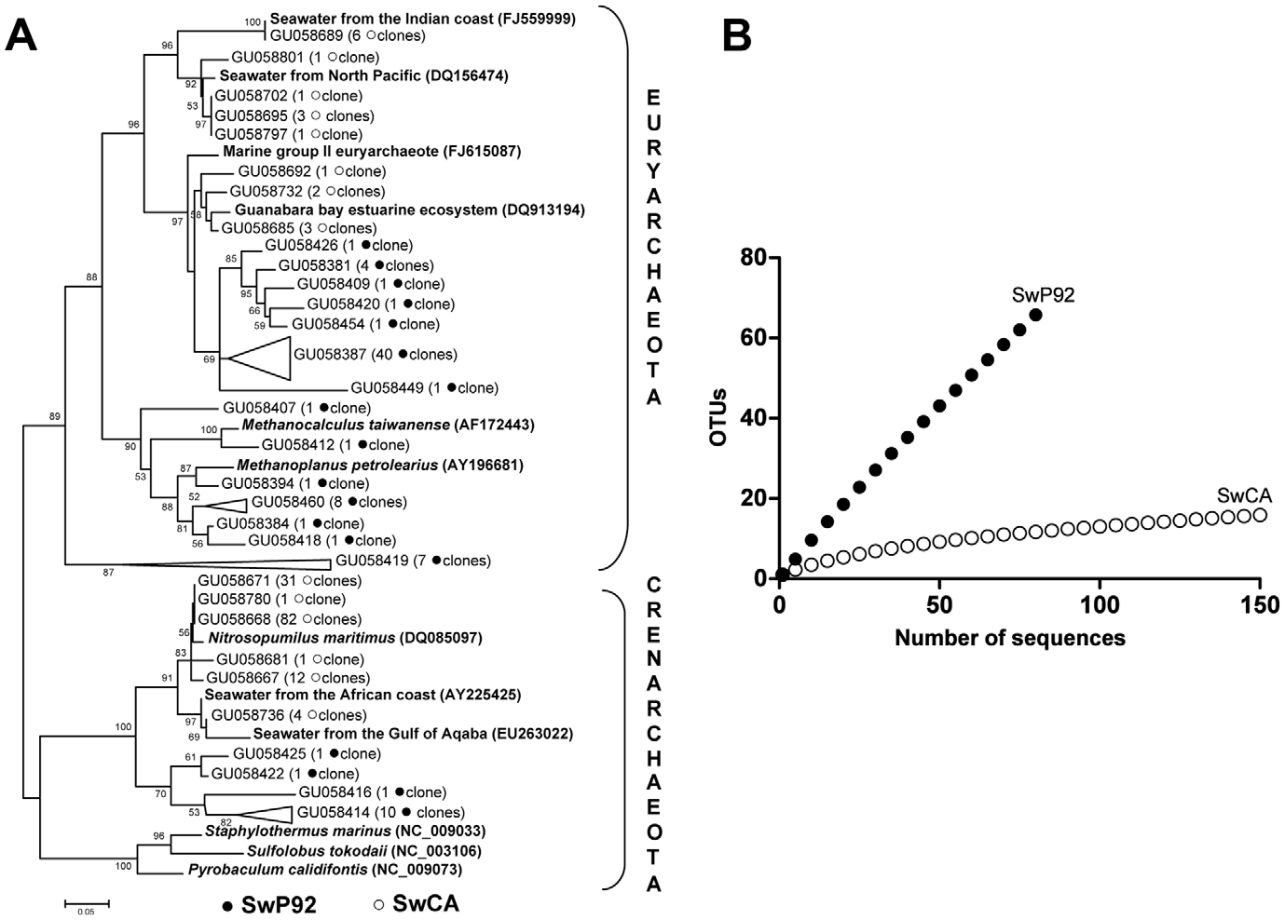
Planktonic archaeal communities. (A) Phylogenetic construction: Neighbour-joining 16S rRNA unrooted tree (•) inner bay (P92) clones (○) Cagarras Archipelago (CA) clones (B) Rarefaction analysis at 97% stringency (•) inner bay (P92) sequences (○) Cagarras Archipelago (CA) sequences.

A total of 235 valid sequences, 85 from inner bay and 150 from the Cagarras Archipelago, with Phred score ≥ 20 were obtained from planktonic samples. These sequences were grouped as OTUs (Operational Taxonomic Units) using DOTUR software based on 97% similarity. Sixty eight OTUs were produced for the inner bay sample while 16 OTUs were observed in the Cagarras sample. Phylogenetic analysis of the archaeal 16S rRNA sequences obtained from these seawater samples showed the presence of the two main archaeal phyla, *Crenarchaeota* and *Euryarchaeota* (Fig. 3A).

Most inner bay clones were representative of *Euryarchaeota*, with many sequences affiliated with clones recovered from previous studies in Guanabara Bay [22]. In addition, some OTUs were closely related to methanogenic archaea such as *Methanoplanus petrolearius* which may come from petroleum polluted anoxic environments around the bay. Based on the current database, BLAST searches performed with some *Euryarchaeota* clusters were only successful in retrieving sequences with low similarity, suggesting that these microorganisms possibly represent a new archaeal group. Most seawater OTUs retrieved from the Cagarras Archipelago were affiliated to environmental uncultured archaeal species. Some OTUs were related to Group II *Euryarchaeota* and also to another archaeon recovered from planktonic archaea from the North Pacific and Indian coasts as shown in the phylogenetic tree (Fig. 3A). Regarding the *Crenarchaeota*, OTUs from the inner bay site did not show high similarities to reference sequences in BLAST searches. On the other hand, OTUs from the Cagarras Archipelago formed a representative cluster (127 clones) related to *Nitrosopumilus maritimus* and to archaea recovered from waters off the African coast and the Gulf of Aqaba.

Rarefaction curves with clusterization at 97% similarity showed higher archaeal species diversity within the bay than in the Cagarras Archipelago seawater (Fig. 3B). The number of clones sequenced from the P92 site was not enough to cover the whole archaeal diversity, while the main archaeal groups were detected in the Cagarras Archipelago. No OTUs were shared between water samples from the two sites.

### Sponge associated Archaea diversity

**Figure 4:**
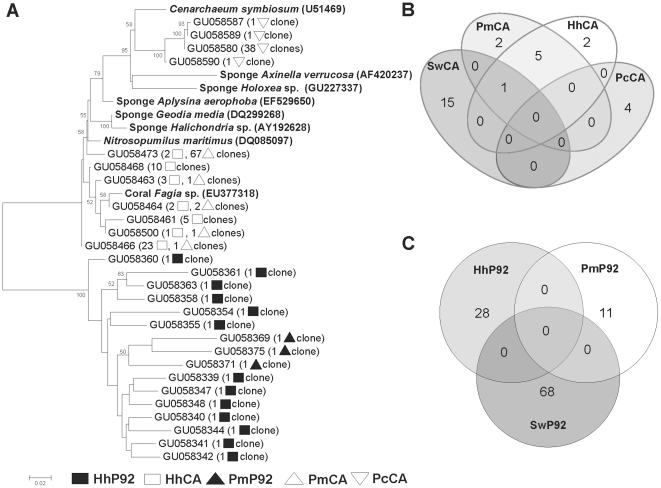
Sponge *Crenarchaeota* communities. (A) Neighbour-joining 16S rRNA phylogenetic tree. Sponge archaeal clones (▪) HhP92, (□) HhCA, (▴) PmP92, (Δ) PmCA and (◊) PcCA Venn diagram with OTUs grouped at 97% similarity in (B) *Archaea* related to seawater and sponges from the Cagarras Archipelago and (C) *Archaea* related to seawater and sponges from P92. Hh, *Hymeniacidon heliophila*; Pm, *Paraleucilla magna*; Pc, *Petromica citrina*; CA, Cagarras Archipelago; P92, inner bay site.

Unlike seawater, phylogenetic analysis of 254 sponge archaeal sequences showed exclusively members of *Crenarchaeota* phylum (Fig. 4A). At 97% stringency, 28 OTUs were produced for the inner bay sponges and eight OTUs were observed in the Cagarras Archipelago sponges. *Archaea* associated with the sponges *H. heliophila* and *P. magna* collected at the Cagarras Archipelago were affiliated to clones retrieved from sponges *Aplysina aerophoba* and *Axinella verrucosa* and corals *Fungia* sp. and *Mussismilia hispida*. Interestingly, all *P. citrina* OTUs clustered together and were closely related to *C. symbiosum*, also described as the sole archaeal symbiont associated with the marine sponge *A. mexicana*. The Venn diagram for the Cagarras Archipelago showed that five OTUs are shared between *P. magna* and *H. heliophila,* and one OTU is shared between both sponges and seawater (Fig. 4B). Regarding the inner bay samples, no OTUs were shared between the sponges or between sponges and the planktonic sample (Fig. 4C).

### Occurrence of archaeal amoA gene in sponges

**Figure 5:**
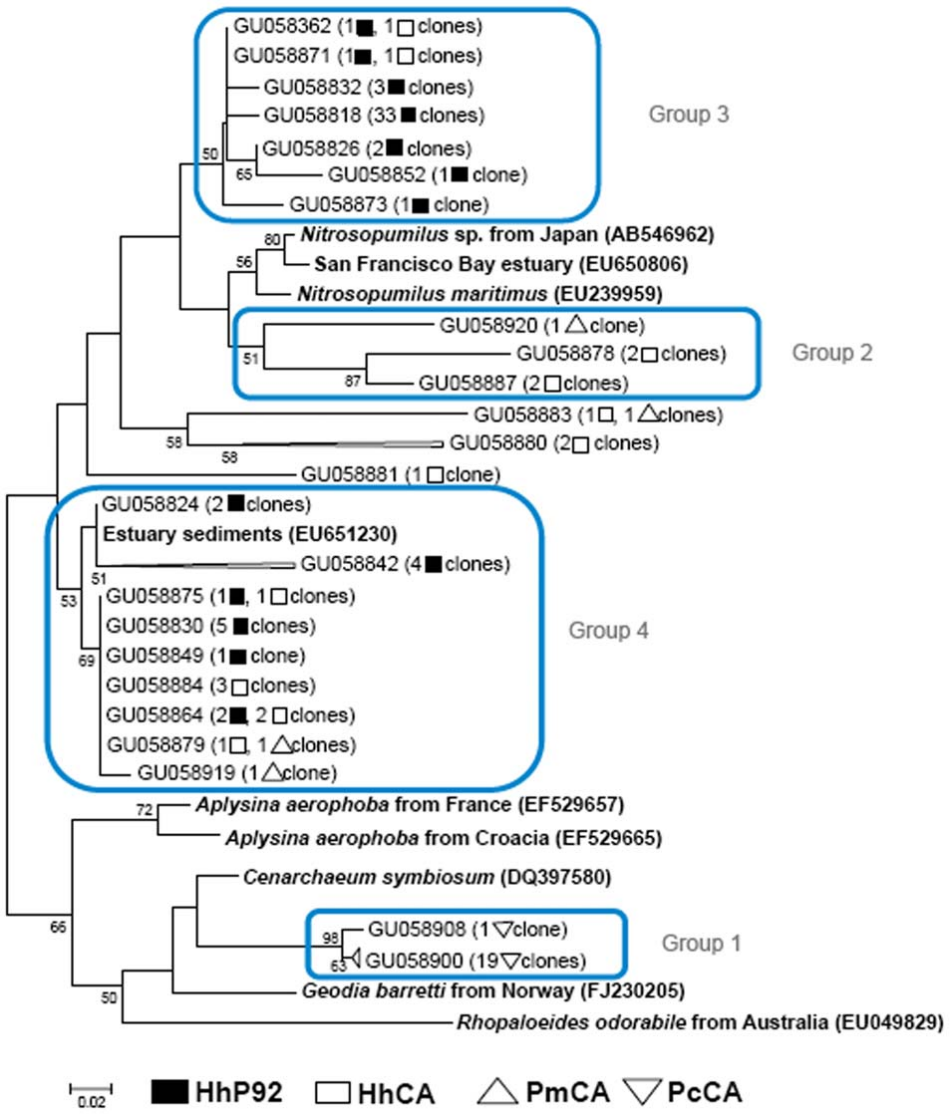
Phylogenetic relationships of sponge archaeal *amoA* genes. Unrooted neighbour-joining phylogenetic tree (▪) HhP92, (□) HhCA, (Δ) PmCA and (◊) PcCA. Hh, *Hymeniacidon heliophila*; Pm, *Paraleucilla magna*; Pc, *Petromica citrina*; CA, Cagarras Archipelago; P92, inner bay site.

Some archaeal OTUs found in our 16S rRNA phylogenetic trees are related to species known for their ammonia oxidizing capacity. Therefore, we constructed a tree of the archaeal *amoA* gene sequences retrieved from the sponges from both sites (Fig. 5). Sequences were separated in four clusters: (1) Twenty sequences related to *C. symbiosium* and exclusively present in *P. citrina*; (2) five sequences related to *N. maritimus* occurring exclusively in *H. heliophila* and *P. magna* from the Cagarras Archipelago; and two broad groups (3 and 4) that combined *H. heliophila* archaeal *amoA* sequences from both sites. Other five clones occurring in *H. heliophila* and *P. magna* from the Cagarras Archipelago are distantly related to the larger group formed by *Nitrosopumilus* and groups 2 and 3.

### Similarities between archaeal communities in sponges and seawater

**Figure 6:**
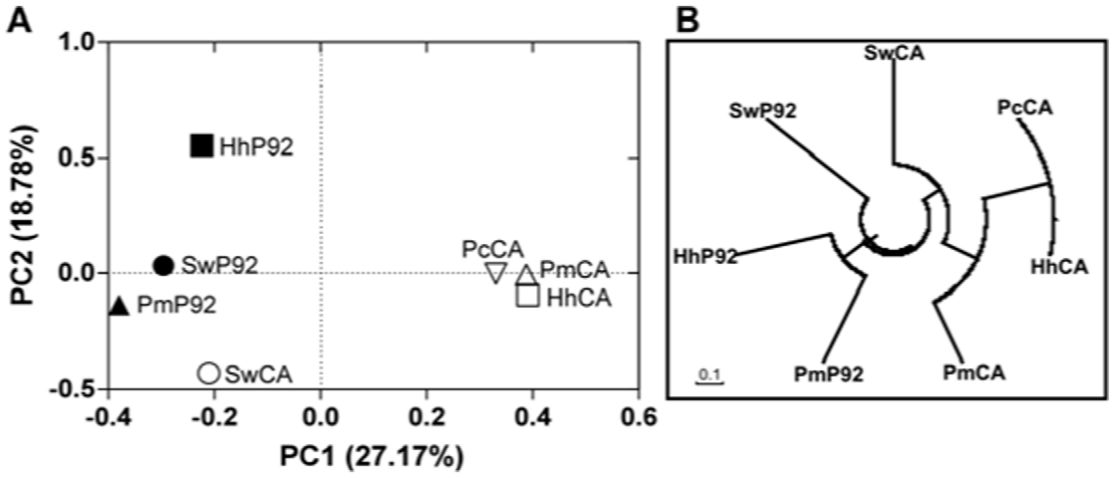
Match between archaeal communities in sponges and seawater samples. (A) Similarity between archaeal communities. Principal coordinates plots (PCA) were generated using the pairwise unweighted UniFrac distances. (B) Community tree showing the similarity of the samples under the Yue & Clayton theta structural diversity measure. Hh, *Hymeniacidon heliophila*; Pm, *Paraleucilla magna*; Pc, *Petromica citrina*; SW, seawater; CA, Cagarras Archipelago; P92, inner bay site.

UniFrac is a beta diversity metric analysis that quantifies community similarity based on phylogenetic relatedness [29]. In order to visualize distribution patterns of archaeal communities we used the UniFrac metric to perform a principal component analysis (PCA) highlighted by significance. In the scatter plot the first two principal components PC1 and PC2 explained 27.17% and 18.78% of data variation, respectively (Fig. 6A). PC1 separated inner bay sponge associated archaeal communities from the Cagarras Archipelago communities. PC2 separated planktonic archaeal communities from the two environments. Community trees can be used to visualize the similarity of different samples. Similar to the PCA results, the community tree (Fig. 6B) suggested that archaeal communities associated with sponges from both inner bay and the Cagarras Archipelago sites were more similar to each other than they were to seawater communities.

## Discussion

Marine pollution causes a general reduction in species richness, diversity and abundance in sponge communities, usually with a few tolerant species becoming dominant in polluted environments [e.g. 30,31]. Our results confirmed the well-known differences in water quality between Guanabara Bay and the coastal region around the Cagarras Archipelago [22], [32]. They also suggest an impact of pollution on sponge communities, with lower richness, diversity and density inside the bay. *Hymeniacidon heliophila* was more abundant inside than outside the bay, whereas *P. magna* was less abundant and *P. citrina* was absent in the inner bay site. These findings indicate that *H. heliophila* and, to a lesser extent, *P. magna* are tolerant and adapted to the eutrophic Guanabara Bay environment, while *P. citrina* seems to be sensitive to such harsh conditions.

The higher planktonic archaeal diversity recorded in Guanabara Bay seawater compared to the Cagarras Archipelago may be a result of the dynamic condition of this estuarine bay with high nutrient levels, different types of pollutants and remarkable water mixture. Seawater in the inner bay site contains mainly sequences affiliated with *Euryarchaeota* members with low identity sequences from the database, possibly representing new species. Moreover, some inner bay OTUs are related to archaea detected in anoxic environments and were similar to sequences detected in a previous study in the same bay [22] and to sequences related to methanogenic archaea [33]. *Crenarchaeota* are well represented and show a lower diversity in planktonic archaea from the Cagarras Archipelago. Sequences in this cluster are closely related to surface water sequences found in the Gulf of Aqaba, a warm marine ecosystem where *Archaea* make up to >20% of the prokaryotic community [34].

The *Crenarchaeota* communities associated with the sponge species studied here were different from those of the surrounding seawater. Specific associations between sponges and *Crenarchaeota* were previously described [6], [8] and support the hypothesis that sponges can select part of their symbiotic microorganisms. In contrast to previous studies that observed the occurrence of *Euryarchaeota* associated with marine sponges [9], [10], we did not observe any *Euryarchaeota* associated with our sponges. Although we found one OTU in common between two sponge species and seawater in the Cagarras samples, it is possible that transient microorganisms coming from seawater were captured by sponge channels. Some *P. magna* associated archaeal OTUs were related to sequences retrieved from marine sediments. They may have been acquired horizontally via incorporation of sediment, as already seen for bacteria in *Polymastia janeirensis* [24]. Interestingly, in *P. citrina*, a sponge species found only in the Cagarras Archipelago, archaeal communities were exclusive to this sponge and were similar to those of *Axinella mexicana* collected in California [13]. The same pattern was observed for the *amoA* gene, where *P. citrina* also harbors an exclusive group of archaea related to *C. symbiosum*. Possibly, the association of *P. citrina* with this archaea cluster is insufficient for the survival of this species in a polluted estuarine environment. However, other sponge species may harbor more diverse and less specific crenarchaeal species, which may improve their fitness in the estuary.

Sponge and seawater samples from seven archaeal clone libraries were sorted into an ordination plot according to phylogenetic community similarity (Fig. 6A). Habitat classification was a strong structuring factor of the archaeal assemblages and communities grouped according to their habitat of origin. Seawater and sponges of the less impacted Cagarras Archipelago were clearly separated from those of the polluted bay environment and its sponges (Fig. 6B). A clustering of environments based on the UniFrac metric showed that these communities were more similar to each other than to the seawater archaeal communities and that the sponges are colonized with distinct clusters of microbial communities according to environmental conditions. Sponges from different oceans contain specific microbial associations [7]. Our results also show that co-specific sponges separated by only a few kilometres contain distinct archaeal communities demonstrating that environmental conditions can modify, directly or indirectly, sponge associated microbial communities to a better-adapted consortium. Such changes may contribute to improve the fitness of sponges living in stressful habitats.

Nitrification (the microbial oxidation of ammonia to nitrite and nitrate) occurs in a wide variety of environments in all oceans and plays a central role in the global nitrogen cycle, although ammonia and nitrite are toxic to most organisms [35]. Ammonia concentration is typically high in eutrophic environments such as Guanabara Bay [32]. The presence of sponge associated ammonia-oxidizing archaea has been observed in different sponge species [7], [13], [15] and could be important for detoxifying sponge tissues and to increase their resistance to eutrophication. In this model, the species with the most adequate or adaptable symbiont community (such as *P. magna* and *H. heliophila*) may survive better and become more abundant in eutrophic environments than sponges without the ability to acquire the appropriate symbionts in polluted areas (such as *P. citrina*). This ability, however, remains to be demonstrated experimentally, as well as the mechanism through which sponges could select their microbial symbionts. Overall, our results suggest one plausible ecological role for the symbiotic relationships of holobiont organisms such as sponges based on the metabolism of ammonia in different archaeal strains.

## Materials and Methods

### Seawater chemistry and microbiology

To address how sponge distribution, sponge associated archaea and archaeaplankton are linked to environmental data, seawater was collected at two sites (Fig. 1A) and analyzed for abiotic (Fig. 1B) and microbiological parameters (Fig. 1C). Chemical data were determined in triplicates by standard oceanographic methods [36]. Temperature, salinity and pH were determined at the moment of sample collection. Ammonia was measured by the indophenol method, nitrite by diazotation, and nitrate by reduction in a Cd-Cu column followed by diazotation. Total phosphorus was evaluated by acid digestion to phosphate, and silicate by reaction with molibdate. Bacterial abundance was analyzed by flow cytometry, in 2 ml water samples that were immediately fixed for 15 min with 2% sterile paraformaldehyde and frozen in liquid nitrogen. At the lab, samples were thawed and analyzed by flow cytometry after nucleic acid staining with Syto13 fluorochrome at 2.5 µM [37], [38]. Bacterial production was estimated by [^3^H]-leucine uptake [39]–[41].

### Seawater and sponge collection

*Hymeniacidon heliophila* (Demospongiae, Halichondriidae) and *Paraleucilla magna* (Calcarea, Amphoriscidae) were collected in April 2008 using SCUBA diving along vertical walls at approximately 10 m. Samples were obtained from the Rio-Niterói bridge at pillar 92 (22°52′14.25″S – 43°09′43.78″W) inside the polluted Guanabara Bay and from the less polluted offshore Cagarras Island (23°01′28.6″S – 043°11′32.7″W) (Fig 1A). *Petromica citrina* (Demospongiae, Halichondriidae) was collected at 16–20 m only on horizontal surfaces at the Cagarra Island since this species is absent from vertical walls and from polluted sites within the bay. All specimens were preserved in 94% ethanol immediately upon collection for further taxonomic characterization and molecular investigation. Five liters of seawater were taken from the sponge collection sites for planktonic archaea library construction.

### Sponge community structure quantification

The sponge communities were sampled from April 2007 to May 2008, using SCUBA diving on vertical walls at 4–20 m. Sponge community structure parameters (Shannon-Wiener diversity, density and species richness) were also estimated, as well as the abundance (ind.m^−2^) and dominance (% of total number of individuals) of *H. heliophila* and *P. magna*, using 20 quadrats (0.25 m^2^) per site. *Petromica citrina* was quantified in August 2010 only on horizontal surfaces between 16–20 m in the Cagarras Island. Significant differences in ecological parameters between the two sites were determined by Student's t test.

### DNA extraction

Three 1 cm^3^ pieces (approximately 400 mg) of each species were collected and pooled. Sponge tissue was dried and ground in a mortar with a pestle. DNA extraction was performed as described by Clementino et al. [42]. DNA was precipitated from the aqueous phase with three volumes of isopropanol overnight at −20°C. Nucleic acids were washed in 70% (v/v) ice-cold ethanol, dried and dissolved in 40 µl water. For further purification we used the DNeasy Tissue Kit according to the manufacturer's instructions (Qiagen GmgH, Hilden, Germany). DNA was quantified by 1% agarose gel electrophoresis. Seawater samples were filtered through a 3 µm pore membrane, which captures colonial and particle-attached microbes, phytoplankton and zooplankton. The free-living planktonic microbes were concentrated on a Sterivex-filter (0.22 µm). DNA extraction was prepared according to Somerville et al. [43], with 50 µL of freshly prepared lysozyme (1 mg/mL) added to filter units containing 1.8 mL of lysis buffer (0.75 M sucrose, 20 mM ethylenediamine tetraacetic acid (EDTA), 50 mM Tris–HCl [pH 8.0]), and the units were incubated at 37°C for 45 min. Then, 50 µL of freshly prepared proteinase K (0.2 mg/mL) and 200 µL of 10% sodium dodecyl sulfate (SDS) were added, and incubated at 55°C for 1 h. Lysates were removed with sterile 3 mL syringes, and the filter units were each rinsed with 1 mL of lysis buffer and incubated for 15 min. The rinse buffer and lysates were pooled and then we performed the phenol-chloroform protocol as previously described [22].

### 16S rRNA and amoA PCR amplification

PCR was performed in 50 µl reaction mixtures (2.5 mM MgCl, 0.2 mM dNTPs, 10 pmol of each primer, 2.5 U of high fidelity Platinum *Taq* DNA polymerase (Invitrogen) and PCR buffer). Approximately 100 ng of genomic DNA was extracted from each sample. To amplify the 16S rRNA gene two oligonucleotides were used: universal prokaryotes reverse primer 907ABR (5′-TTTGAGTTTMTTAATGCC-3′) [44], and universal *Archaea* forward primer 21AF (5′-TTCCGGTTGATCCTGCCGGA-3′) [11]. PCR amplification began with a 5 min denaturing step at 94°C; this was followed by 30 cycles at 94°C for 1.30 min, 50°C for 1.30 min, and 72°C for 2 min. The final cycle was an extension at 72°C for 10 min. PCR products were purified with GFX PCR DNA and gel band purification kit following the manufacturer's instructions (GE, Healthcare). The *amoA* gene fragment was obtained using the primer pair described by Francis et al. [35] and amplification was performed according to the protocol of Steger et al. [17].

### Archaeal gene library construction

Two archaeal 16S rRNA gene libraries were constructed from free-living planktonic microbe samples and five from marine sponges, for the two environments, Guanabara Bay and Cagarras Archipelago. Construction of the *amoA* gene library was performed only with *H. heliophila* from the inner bay site, while for the Cagarra Island three species were used: *H. heliophila, P. magna* and *P. citrina*. PCR fragments were cloned into pGEM-T cloning vector (Promega) and used to transform *E. coli* DH10B electro-competent cells.

### Sequence analyses

DNA from each clone was prepared and sequences were obtained by cycle sequencing with the Big Dye reagent (Applied Biosystems, Foster City, CA) and then analyzed in an Applied Biosystems ABI Prism 3730 automated DNA sequencer [45]. Sequences with approximately 880 bp were obtained using 21F primer and those with less than 300 bp and chimeras were removed. NCBI BLAST searches were performed to identify the nearest neighbor. Alignments with representative archaeal sequences obtained at GenBank databases were carried out using ClustalX [46]. Sequences were clustered as Operational Taxonomic Units (OTUs) using DOTUR [47]. OTUs of 16S rRNA and *amoA* genes were defined as groups in which sequences differed by 3 and 5%, respectively. Diversity of archaeal phylotypes was further examined using rarefaction analysis [48], [49]. Phylogenetic trees were constructed by neighbour-joining [50] based on distance estimates calculated by the Kimura-2 algorithm [51]. Tree construction was performed with MEGA4 [52] and ARB [53]. Tree topology and distribution of hits along the tree were uploaded to UniFrac online computational platform [29], [54]. Venn diagrams, rarefaction analysis, check chimera and community trees were made using MOTHUR [55]. To generate a community tree we used a newick-formatted tree that indicates how similar our samples are according to the Yue & Clayton theta structural diversity measure as described in MOTHUR manual. Partial 16S rRNA and *amoA* archaeal sequences generated in this study have been deposited in GenBank, Accession Numbers GU058339-GU058920.

### Ethics statement

All animal work was conducted according to relevant national and international guidelines. Samples were collected under a Scientific Research Permit issued by the Instituto Brasileiro de Meio Ambiente e Recursos Renováveis (IBAMA), of the Brazilian Government.
